# Effects of Long-term low-dose intermittent rapamycin administration on glucose metabolism and immune system of SAMP8 and SAMR1 mice

**DOI:** 10.3389/fimmu.2025.1682406

**Published:** 2025-10-21

**Authors:** Luiz Adriano Damasceno Queiroz, Rafael Santos Barros, Josiane Betim Assis, Camille Cristine Caldeira Silva, Walter Miguel Turato, Sofia Xavier Bustia, Stephen Fernandes Rodrigues, Anderson Sá-Nunes, Joilson O. Martins

**Affiliations:** ^1^ Laboratory of Immunoendocrinology, Department of Clinical and Toxicological Analyses, School of Pharmaceutical Sciences - São Paulo University, São Paulo, Brazil; ^2^ Laboratory of Experimental Immunology, Department of Immunology, Institute of Biomedical Sciences - São Paulo University, São Paulo, Brazil; ^3^ Energy Metabolism Laboratory, Department of Biochemistry, Institute of Chemistry - University of Sao Paulo, São Paulo, Brazil; ^4^ Multi-User Preclinical Imaging Center, Department of Clinical and Toxicological Analyses, School of Pharmaceutical Sciences - São Paulo University, São Paulo, Brazil; ^5^ Laboratory of Vascular Nanopharmacology, Department of Pharmacology, Institute of Biomedical Sciences of University - Sao Paulo, Sao Paulo, Brazil

**Keywords:** rapamycin, SAMR1, SAMP8, immunosenescence, mitochondria

## Abstract

Aging involves a gradual decline in physiological integrity, and rapamycin (RAPA) has demonstrated potential as an anti-aging agent. Nonetheless, its effects on glucose metabolism and immune function may vary based on dosage and administration regimen. This study investigates the impact of intermittent low-dose RAPA on glucose metabolism and immune function in Senescence-Accelerated Mouse Prone 8 (SAMP8) and Senescence-Accelerated Mouse Resistant 1 (SAMR1) mice. Twelve-week-old male SAMP8 and SAMR1 mice were treated with RAPA (0.78 µg/kg) every five days for six months. Glucose uptake, mitochondrial respiratory capacity, spleen and thymus immunophenotype, lymphoproliferation, and cytokine profiles were evaluated. Our findings indicate that RAPA reduced glucose uptake in the bladder and the percentage of FoxP3^+^ lymphocytes in the spleen of SAMP8 mice, while enhancing mitochondrial respiratory control and ATP production in liver. In SAMR1 mice, RAPA administration led to a decrease in CD3^+^ thymocytes and splenic lymphoproliferative capacity, while also enhanced mitochondrial performance. Comparisons between Control groups revealed that SAMP8 mice exhibited higher glucose uptake in several tissues, lower lymphocyte populations in spleen and thymus, altered CD4^+^/CD8^+^ ratios, and reduced IL-4 expression compared with SAMR1 mice. The findings reinforce the potential of RAPA to modulate aging-related processes, highlighting improvements in mitochondrial function and energy metabolism across strains with different aging processes. However, the immunosuppressive effects of RAPA remain evident, even at low doses administered intermittently, in an age- and strain-specific manner. These findings emphasize the therapeutic potential of RAPA while underscoring the need for customized dosing strategies to balance efficacy and safety. These data highlight mitochondrial metabolic improvements as the primary benefit of intermittent low-dose RAPA and suggest potential clinical relevance for conditions involving compromised mitochondrial energy metabolism.

## Graphical Abstract

This study evaluated how rapamycin treatment affects hepatic metabolism and immunological parameters in SAMR1 and SAMP8 mice. We measured *in vivo* glucose uptake, hepatic mitochondrial function, and lymphocyte profiles in the thymus and spleen. We found that the treatment increased mitochondrial capacity in the liver and affected the profile of regulatory T cells. In untreated animals, differences were identified in lymphocyte populations in the spleen and thymus, as well as in glucose uptake in muscle, liver, heart, and bladder in the SAMP8 model (characterized by accelerated senescence) compared with the SAMR1 control group.The abstract should ideally be structured according to the IMRaD format (Introduction, Methods, Results and Discussion). Provide a structured abstract if possible. If your article has been copyedited by us, please provide the updated abstract based on this version.

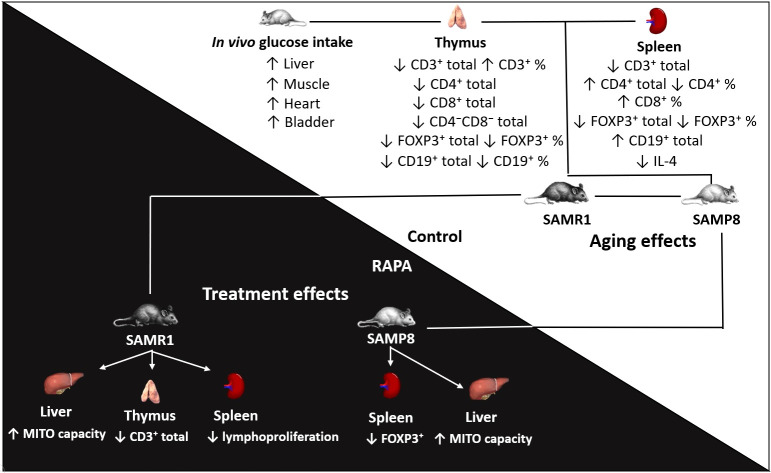

## 1 Introduction

Aging is a natural process characterized by the gradual accumulation of cellular defects, which progressively impair proliferative capacity of cells and disrupt essential functions required for organismal survival (1). During the aging process, defective cells that are not eliminated often transition into a senescent phenotype, marked by irreversible cell cycle arrest while maintaining metabolic activity (2, 3). Over time, an increasing number of cells adopt this senescent phenotype, accumulating in tissues and organs, thereby impairing homeostasis and regeneration (4). This accumulation arises not only from reduced proliferative capacity but also from the secretion of senescence-associated secretory phenotype (SASP) inflammatory factors, such as IL-1β, IL-6, and TNF-α. Together, these factors contribute to a state of chronic, low-grade inflammation known as inflammaging, which is implicated in the pathogenesis of common age-related conditions such as cardiovascular disease and diabetes (5–7). To maintain homeostasis, cells rely on various repair mechanisms, with autophagy, a cellular degradation process regulated by the mTOR (or mechanistic target of rapamycin) receptor playing a crucial role (1, 8). Specifically, mitophagy, the autophagic clearance of mitochondria, is essential to preserve mitochondrial function during aging, ensuring the bioenergetic homeostasis necessary for optimal cellular function (1, 9, 10). The accumulation of dysfunctional mitochondria is a hallmark of aging (10–12). In fact, mitochondrial homeostasis is vital not only for cellular metabolism but also for overall health. Mutations in mitochondrial DNA are associated with a range of adverse phenotypes, including myopathies, neuropathies, diabetes, premature aging, and shortened lifespan. Mitochondrial dysfunction is a common feature of aging, and mTOR downregulation promotes mitochondrial biogenesis and maintenance, thereby contributing to longevity (12).

Aging also fosters a vicious cycle between inflammation and immunosenescence. Increased levels of inflammatory mediators reduce adaptive immune responses, further promoting immunosenescence. As a result, the innate immune cells intensify their functions, releasing more pro-inflammatory mediators, which in turn sustain the low-grade inflammation typical of aging (13).

The discovery that mTOR inhibition can alleviate various aspects of aging and extend lifespan across multiple species has fueled scientific interest in developing mTOR inhibitors to potentially enhance human longevity (1, 8). Rapamycin (RAPA), a well-studied mTOR inhibitor isolated from *Streptomyces* strains, has shown promise as an immunosuppressant and autophagy inducer. RAPA induces a nutrient-starvation response, enabling cells to conserve resources, which delays the onset of cellular senescence and extends lifespan (2, 9, 14). In aged mice, RAPA treatment doubles B lymphocyte counts in bone marrow, enhancing viral infection response and vaccination efficacy (15). Furthermore, RAPA treatment increases the levels of regulatory T (Treg) cells in the spleen and thymus without impairing their function *in vitro* and/or *in vivo*, though it does suppress the CD4^+^ T cell response (16).

Despite its considerable potential, extended use of RAPA can lead to clinical complications, including glucose metabolism disruption, insulin resistance, gonadal atrophy, and cataract formation (15, 17, 18). Additionally, RAPA also improves mitochondrial homeostasis, enhancing metabolic stress adaptation during replicative aging *in vitro* and *in vivo*. This adaptation encompasses increased mitochondrial carbon oxidation, maintaining ATP levels and redox balance for bioenergetic requirements of cellular maintenance and proliferation (2). RAPA, by inhibiting mTORC1, indirectly activates the PINK1/Parkin pathway and other mitophagy-related proteins such as BNIP3 and NIX, promoting the recognition and removal of dysfunctional mitochondria and thereby supporting mitochondrial quality Control (19–21).

Replicative senescence often manifests as a metabolic shift from glycolysis to oxidative phosphorylation, despite increased mitochondrial mass and activity markers, likely due to lysosomal pH elevation from proton pump failure, impairing cellular organelle clearance. The buildup of dysfunctional mitochondria in senescent cells amplifies the production of reactive oxygen species (ROS), leading to cellular damage, with mTOR being central to these metabolic changes. Consequently, inhibition of mTOR by RAPA mitigates metabolic stress and delays the onset of senescence (1).

Negative mTOR regulation, via caloric restriction or RAPA inhibition, has been shown to extend lifespan across a wide range of species, including fungi (22), nematodes (23), arthropods (24), rodents (25), and non-human primates (26). This longevity effect is primarily attributed to induction of autophagy, a vital process that rejuvenates cells (27), maintains protein and mitochondrial homeostasis (22, 28), ensures genetic stability (29), and downregulates inflammasome activity (2, 30). These findings underscore the evolutionary significance of mTOR as a longevity regulator (1).

Aging represents a significant and growing public health concern, underscoring the urgent need for innovative therapeutic strategies aimed at enhancing the well-being of older populations. While mTOR inhibition via RAPA has shown promising anti-aging benefits, its long-term application is hindered by adverse effects on immune function and glucose homeostasis. To address this critical gap, our study evaluates the effects of intermittent low-dose RAPA administration on glucose metabolism, immune modulation, and mitochondrial function in Senescence-Accelerated Mouse Prone 8 (SAMP8) and Senescence-Accelerated Mouse Resistant 1 (SAMR1) mouse models. This approach seeks to maximize the therapeutic potential of RAPA while mitigating its systemic side effects, advancing the development of safer anti-aging interventions.

## 2 Material and methods

### 2.1 Animals and RAPA administration

Twelve-week-old male SAMP8 and SAMR1 mice were used. The animals were maintained at 23 ± 2°C, in mini-isolators, in a 12-hour light/dark cycle, with water and food *ad libitum*. For anesthesia prior to euthanasia, mice were exposed to isoflurane at 4% in a closed chamber until loss of reflexes, followed by cervical dislocation to ensure death. All experiments were conducted in strict accordance with the principles and guidelines of the National Council for the Control of Animal Experimentation (CONCEA) and approved by the Ethics Committee on Animal Use (CEUA) at the School of Pharmaceutical Sciences of the University of São Paulo, Brazil (protocol number: CEUA/FCF/619).

RAPA (InvivoGen, Toulouse, France) was resuspend in dimethyl sulfoxide (DMSO Sigma-Aldrich, St. Louis, MO, USA), following the manufacturer’s recommendations. The dose to be administered was calculated based on the animal’s weight. In the treated group animals, we administered a dose of 0.78 µg/kg of RAPA per mouse diluted in 150 µL of filtered water. The Control group animals received only vehicle solution (filtered water) (31). The administration was performed by gavage, once every 5 days, for 6 months. The gavage procedure was typically performed between 2 and 4 p.m., and samples were collected the day after the last dose at 8 a.m.

### 2.2 Assessment of glucose metabolism *in vivo*

Initially, blood glucose levels were measured using an Accu-Chek Active^®^ glucose monitor after a 12-hour fasting period. Subsequently, the animals were anesthetized with isoflurane (2–3% in medical-grade oxygen) delivered via a nose cone, and 22.2 MBq (600 μCi) fluorine 18-labeled fluorodeoxyglucose (18F-FDG) was intravenously administered in 100 μL of phosphate-buffered saline (PBS). One hour post-injection (biodistribution period of the radiotracer), the animals were re-anesthetized with isoflurane (1.5%) via an oxygen mask to maintain anesthesia during the acquisition of PET scans. The animals were positioned on the imaging bed, and PET scans were acquired using the Albira PET-SPECT-CT scanner (Bruker Biospin, Valencia, Spain) for small animal studies. The static PET image was acquired for 1 hour with 94.4 mm. Immediately following PET acquisition, a CT scan was performed using 400 projections at 45 kVp and 400 μA, with a magnification factor of 1.46. Post-acquisition, PET images were reconstructed using the maximum-likelihood expectation-maximization (MLEM) algorithm with 12 iterations. Corrections were applied for radioactive decay, scatter, and random events; however, attenuation correction was not performed. CT images were reconstructed using filtered back projection (FBP) algorithm. The resulting images were analyzed using PMOD software (PMOD Technologies, Zurich, CH) to quantify glucose uptake in the muscle, liver, heart, kidneys, and bladder. The 18F-FDG uptake was expressed as a standardized uptake value (SUV), calculated as the radioactivity concentration (kBq/cc) normalized to the ratio of injected dose (kBq) to the animal’s body weight (g) (32).

### 2.3 Oxygen consumption in isolated liver mitochondria

Liver mitochondria were isolated following the hepatic mitochondria separation protocols, as previously described by Tahara et al. (2009) (33). Briefly, liver tissue was processed in an isolation buffer containing 250 mM sucrose, 10 mM HEPES, and 1 mM EGTA (pH 7.2, adjusted with KOH). Mitochondria were separated from the samples by differential centrifugation: liver homogenate was centrifuged at 600 g for 5 min, and the resulting supernatant was further centrifuged at 12,000 *g* for 10 min. The resulting pellet was resuspended and centrifuged again at 7,000 *g* for 3 min. The isolated mitochondrial sediment was then resuspended in a buffer containing 250 mM sucrose, 10 mM HEPES, and 1 mM EGTA (pH 7.2, adjusted with KOH) and kept on ice for up to 4 hours for oxygen consumption analysis.

Mitochondrial oxygen consumption was monitored in samples containing 50 mg of mitochondrial protein per mL of suspension, using the Clark-type electrode apparatus (Oroboros Instruments, Innsbruck, Austria). To measure State 3 respiration, 1 mM ADP was added, while 1.5 μg/mL oligomycin was used to determine oligomycin-insensitive respiration. Maximal respiration was promoted by incremental additions of 0.5 μM CCCP. The respiratory Control ratios were derived as the ratio of State 3 (ADP- stimulated) to oligomycin rates (34).

Mitochondria were isolated in the morning and the entire extraction procedure was completed by early afternoon. High-resolution respirometry assays were performed between 2 and 5 p.m on the same day of isolation. During this interval, samples were kept on ice in respiration buffer and protected from oxygenation to minimize metabolic activity. The functional integrity of each preparation was verified before experimental measurements by determining the respiratory control ratio (RCR) under state 3 and state 4 conditions, and only preparations with RCR ≥ 5 were accepted for analysis.

### 2.4 Immunophenotyping by flow cytometry

Following euthanasia, spleen and thymus were removed and macerated in a 40 μm cell strainer. Then, the erythrocytes were lysed using ACK Lysing Buffer (Gibco, Grand Island, NY, USA). The resulting cells were suspended at 2 × 10^6^ cells/mL in PBS and incubated at 4°C for 10 min with LIVE/DEAD™ Fixable Aqua Dead Cell Stain Kit (Invitrogen, Carlsbad, CA, USA) for viability evaluation. Then, cells were incubated for 30 min with the following fluorochrome-conjugated monoclonal antibodies: CD16/CD32 (Fc block, BD Biosciences, clone 2.4G2), CD3 (FITC, BioLegend, clone 145-2c11), CD4 (APC-Cy7, BD Biosciences, clone GK1.5), CD8 (PE-Cy5, BD Biosciences, clone 53-6.7), CD19 (APC, BioLegend, clone 1D3), CD25 (Pacific Blue, BioLegend, clone PC61), and FOXP3 (PE, BioLegend, clone FJK-16s). The cells were rinsed, then fixed and permeabilized using the FoxP3/Transcription Factor Staining Buffer Set (eBioscience, Carlsbad, CA, USA). After staining for 1 hour with anti-FoxP3-PE according to the manufacturer’s instructions, the samples were washed and resuspended in PBS with 2% fetal bovine serum (FBS). The cells were transferred into polypropylene tubes (12 × 75 mm), acquired using the Cytek Northern Lights flow cytometer (Cytek^®^ Biosciences, Fremont, USA), and analyzed using FlowJo v7.5.5 software (BD Life Sciences). Dot-plot gating was applied based on forward scatter (FSC) and side scatter (SSC) to identify lymphocytes, excluding doublets and dead cells (**Supplementary Figure 1**) (35).

### 2.5 Analysis of lymphoproliferative response

A suspension containing 1.25 × 10^5^ spleen cells/mL was prepared and distributed into 96-well flat bottom plates. Cells were cultured in complete medium only (Control) or stimulated with 1 µg/mL concanavalin A (ConA). The plates were maintained in a CO_2_ incubator at 37°C for 72 hours. After the first 48 hours of culture, 25 µL of 0.01% resazurin was added to the wells, and at the end of 72 hours, the absorbance was analyzed as previously described (35).

### 2.6 Cytokine analysis in lymphoproliferative supernatants

For cytokine quantification, parallel lymphoproliferation cultures were established using a suspension of 5 × 10^6^ spleen cells/mL, plated at 100 μL per well in 96-well flat-bottom plates and stimulated as described above. After 72 hours, cell-free supernatants were collected for quantification of IFN-γ, IL-4, and IL-5 using OptEIA™ ELISA kits (BD Biosciences, San Diego, CA, USA), following the manufacturer’s protocol. Cytokine values (pg/mL) were determined using standard curves from recombinant proteins, with respective detection limits of 7.8 pg/mL (IL-4), 15.6 pg/mL (IL-5), and 31.3 pg/mL (IFN-γ).

### 2.7 Statistical analysis

The results were evaluated by analysis of variance (ANOVA), followed by Tukey’s multiple comparison test, using the GraphPad Prism 6.0 software program (GraphPad Software, San Diego, CA, USA). The normality of data distribution was verified using the Shapiro–Wilk test. Data were represented as mean ± standard error, and values of p < 0.05 (indicated as * or #) were considered significant.

## 3 Results

### 3.1 SAMP8 and SAMR1 mice displays distinct profiles of glucose metabolism *in vivo*

**Figure 1:**
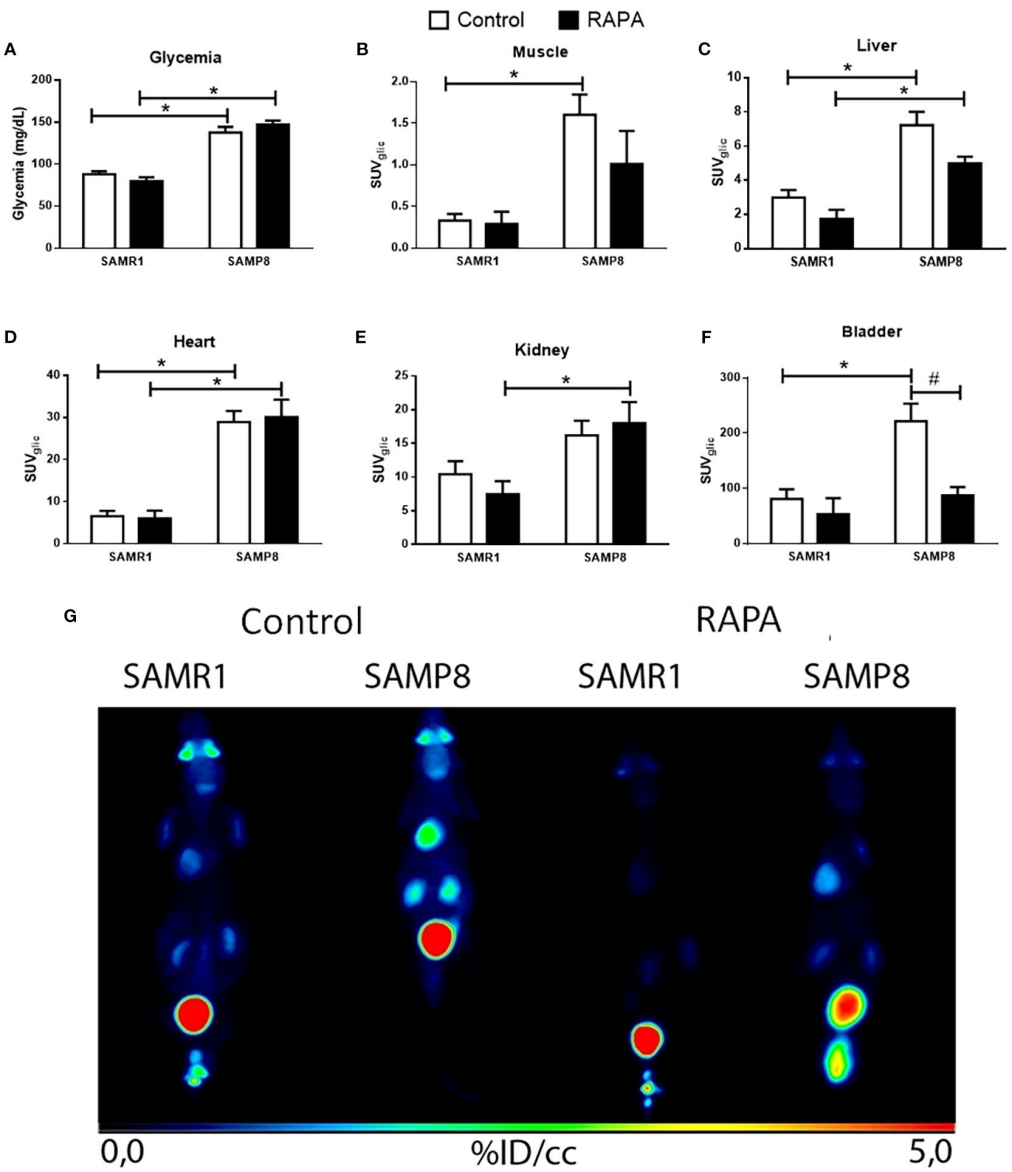
Organ-specific *in vivo* glucose metabolism. **(A)** Blood. **(B)** Glucose uptake in the muscle; **(C)** Liver; **(D)** Heart; **(E)** Kidneys; **(F)** Bladder; **(G)** Photo of representative image of PET-CT. Results are expressed as mean ± standard error. Statistical analysis was performed using a two-way ANOVA with two fixed factors strain (SAMR1 vs. SAMP8) and treatment (Control vs. RAPA) followed by Tukey’s multiple-comparison test. *indicates a significant difference between Control strains, and # indicates a significant difference between SAMP8 groups (p ≤ 0.05). (n=5 animals per group). Raw data on **Supplementary Table S1**.

Evaluation of systemic glycemia demonstrated a significant elevation in blood glucose levels in SAMP8 mice relative to SAMR1, both in the Control and RAPA groups, reflecting the characteristic hyperglycemic phenotype of the SAMP8 strain (**Figure 1A**). RAPA treatment did not significantly alter glycemia in either strain. Analysis of tissue-specific glucose uptake in Control animals revealed that SAMP8 Control demonstrated markedly increased glucose uptake in the muscle, liver, heart, and bladder compared to SAMR1 Control (**Figures 1B–D, F**), indicative of an altered metabolic state.

In RAPA-treated animals, lineage-dependent differences persisted in certain organs, such as the liver and heart (**Figures 1C, D**), where SAMP8 RAPA continued to display higher glucose uptake compared to SAMR1 RAPA. However, these differences were attenuated or lost in other organs, namely muscle and bladder, where no significant differences remained between strains following treatment. Notably, in the kidney, a difference between groups emerged only after RAPA treatment, with SAMP8 RAPA showing higher glucose uptake than SAMR1 RAPA (**Figure 1E**).

When comparing the Control and treated groups within each strain, we observed a lower ^18^F-FDG signal in the bladder of SAMP8 animals treated with RAPA (**Figure 1F**). Since the bladder does not metabolize glucose but rather accumulates and excretes non-metabolized ^18^F-FDG, this finding should be interpreted with caution. It may reflect differences in urine production, renal excretion, or bladder emptying during imaging, rather than a direct effect on glucose metabolism.

### 3.2 RAPA enhances ATP synthesis in both SAMP8 and SAMR1 strains

**Figure 2:**
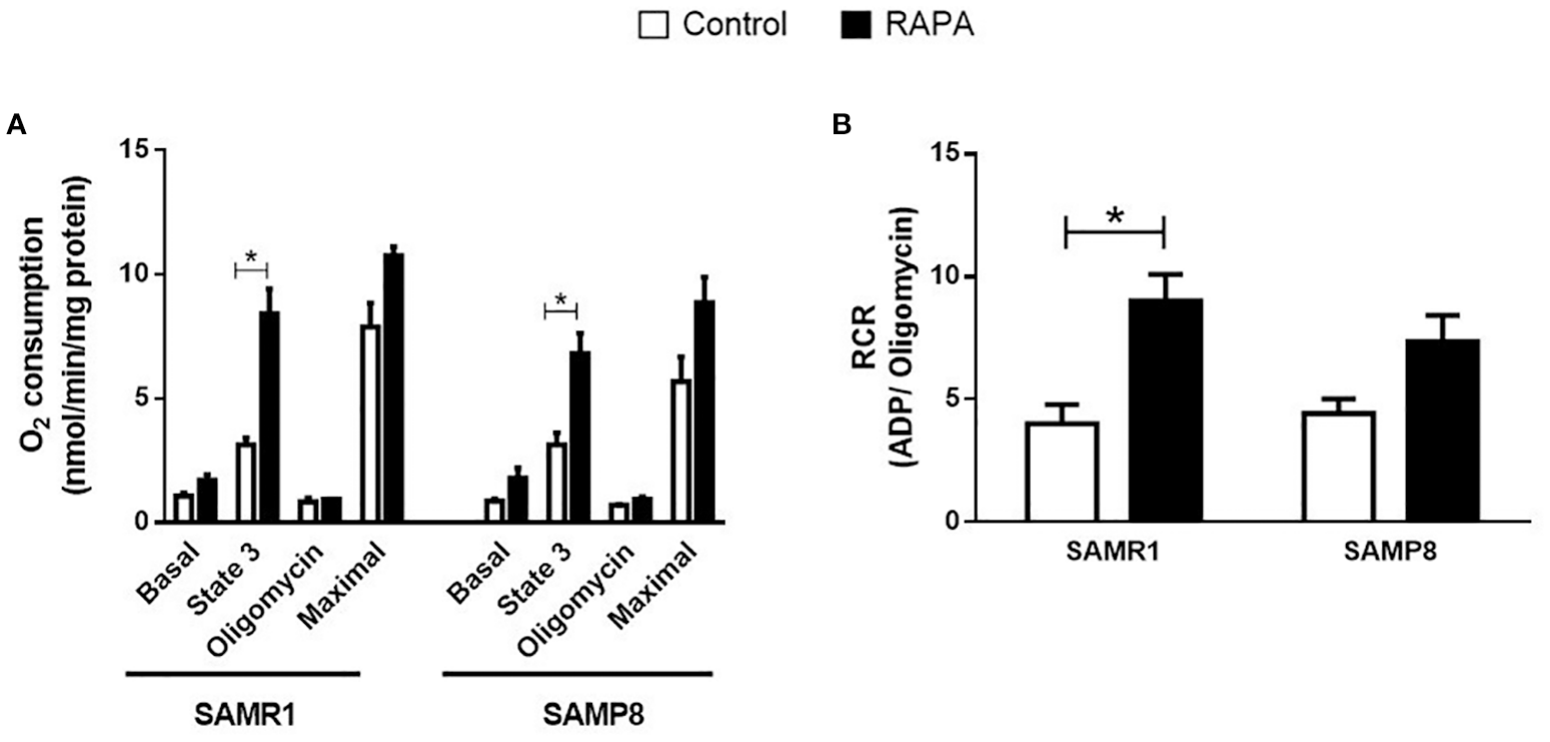
Respiratory capacity of mitochondria isolated from the liver. **(A)** Basal capacity; **(B)** Respiratory Control. Results are expressed as mean ± standard error. Statistical analysis was performed using a two-way ANOVA with two fixed factors strain (SAMR1 vs. SAMP8) and treatment (Control vs. RAPA) followed by Tukey’s multiple-comparison test. *indicates a significant difference between Control strains, and # indicates a significant difference between SAMP8 groups (p ≤ 0.05). (n=3–4 animals per group). Raw data on **Supplementary Table S2**.

Liver mitochondrial respiratory capacity was assessed using a Clark-type electrode following 6 months of RAPA treatment. Both SAMP8 and SAMR1 strains treated with RAPA exhibited enhanced ATP synthesis, as evidenced by a significantly increased response to ADP stimulation compared to their respective Controls (**Figure 2A**). Furthermore, SAMR1 mice demonstrated a marked improvement in respiratory Control ratio, indicating a more efficient coupling of mitochondrial respiration under RAPA treatment (**Figure 2B**).

### 3.3 RAPA negatively affects populations of T lymphocytes in spleen and thymus

**Figure 3:**
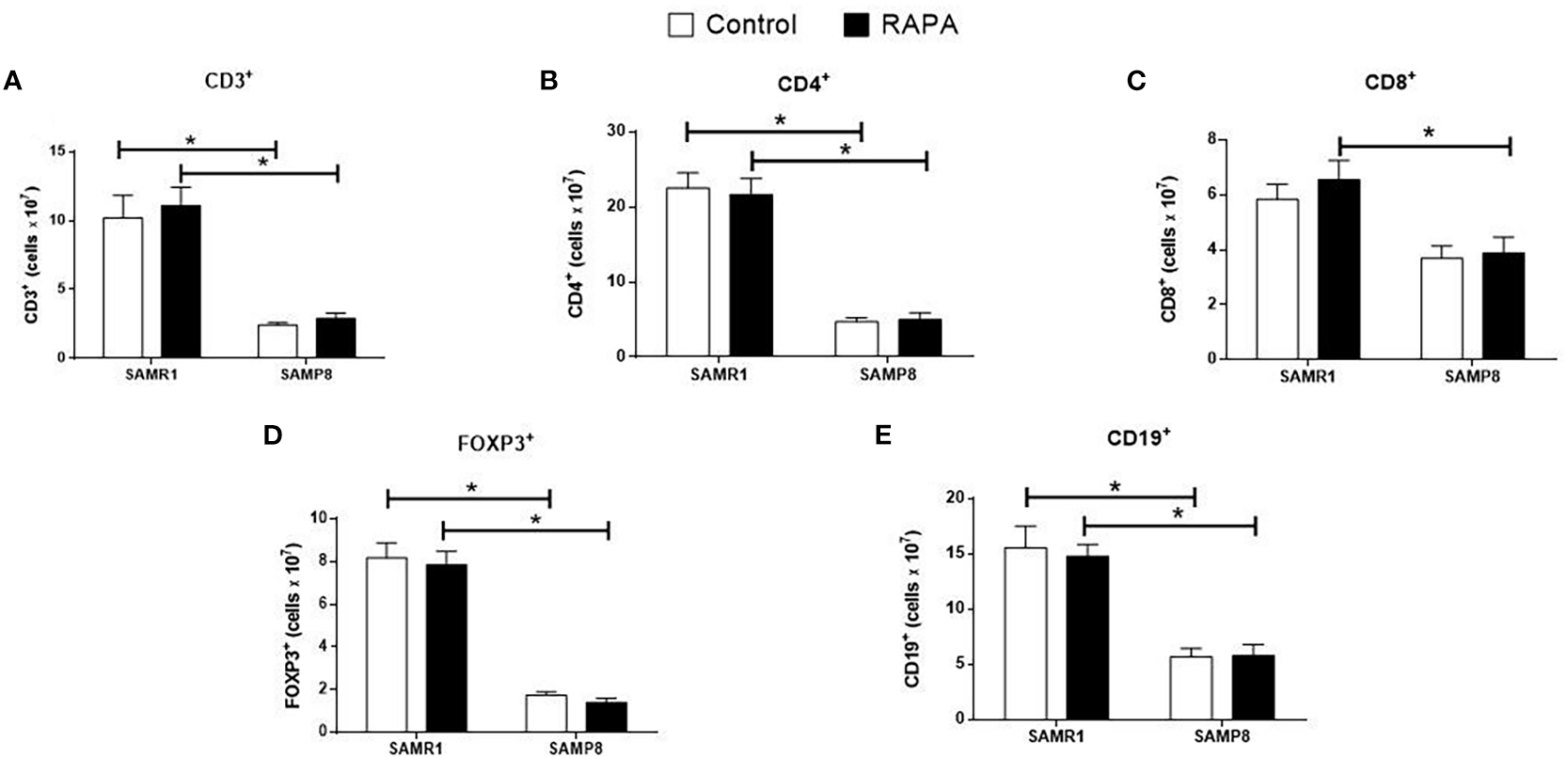
Assessment of the total number of lymphocyte population in the spleen of animals after 6 months of RAPA treatment. T lymphocytes: **(A)** Total lymphocytes (CD3^+^ cells); **(B)** T helper lymphocytes (CD4^+^ cells); **(C)** Cytotoxic T lymphocytes (CD8^+^ cells); **(D)** Regulatory T lymphocytes (FoxP3^+^ cells); B lymphocytes: **(E)** CD19^+^ cells. Results are expressed as mean ± standard error. Statistical analysis was performed using a two-way ANOVA with two fixed factors strain (SAMR1 vs. SAMP8) and treatment (Control vs. RAPA) followed by Tukey’s multiple-comparison test. *indicates a significant difference between Control strains, and # indicates a significant difference between SAMP8 groups (p ≤ 0.05). (n= 4–5 animals per group). Raw data on **Supplementary Table S3**.

**Figure 4:**
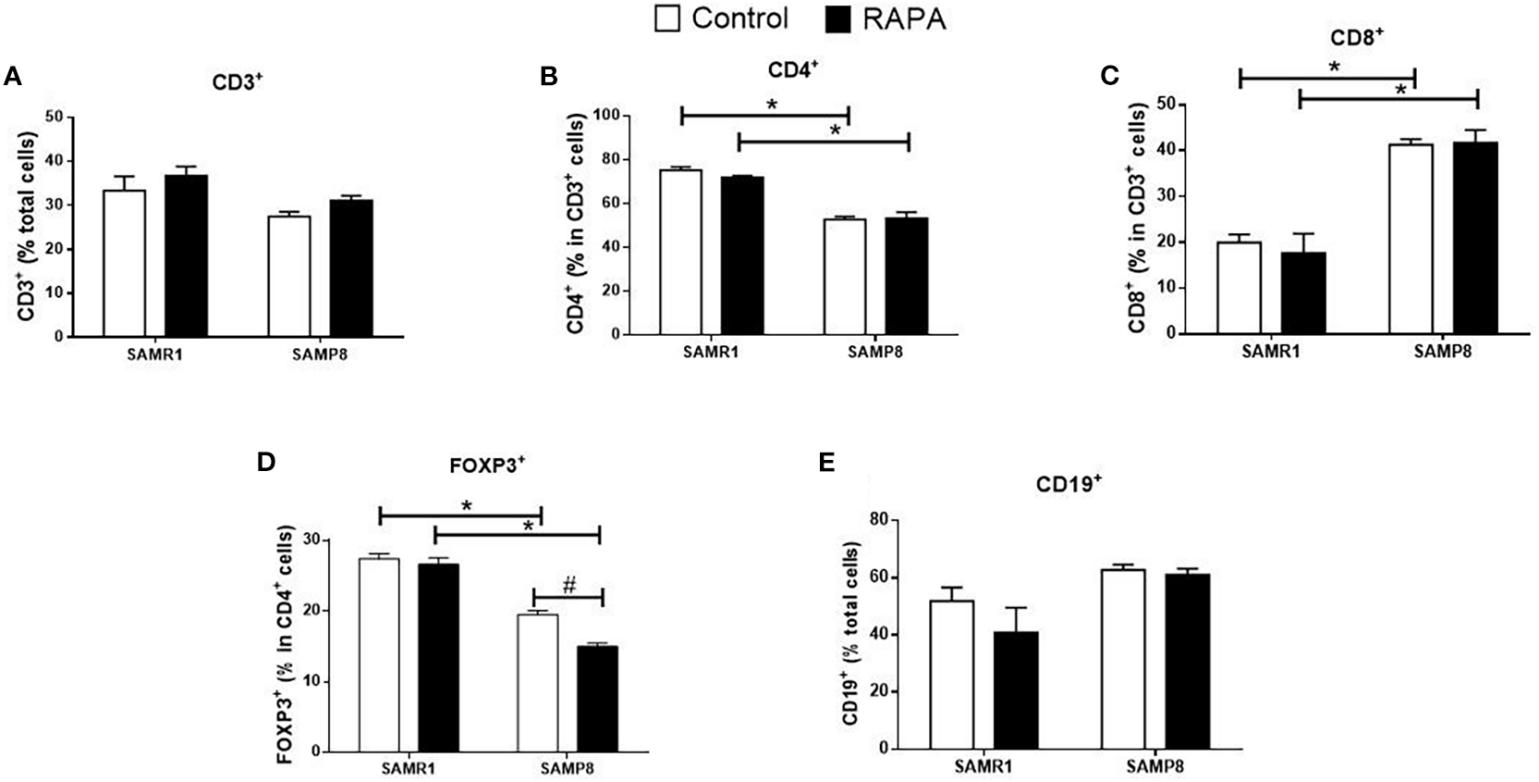
Assessment of the percentage of lymphocyte population in the spleen of animals after 6 months of RAPA treatment. T lymphocytes: **(A)** Total lymphocytes (CD3^+^ cells); **(B)** T helper lymphocytes (CD4^+^ cells); **(C)** Cytotoxic T lymphocytes (CD8^+^ cells); **(D)** Regulatory T lymphocytes (FoxP3^+^ cells); B lymphocytes: **(E)** CD19^+^ cells. Results are expressed as mean ± standard error. Statistical analysis was performed using a two-way ANOVA with two fixed factors strain (SAMR1 vs. SAMP8) and treatment (Control vs. RAPA) followed by Tukey’s multiple-comparison test. *indicates a significant difference between Control strains, and # indicates a significant difference between SAMP8 groups (p ≤ 0.05). (n=4–5 animals per groups). Raw data on **Supplementary Table S4**.

RAPA treatment resulted in lineage-dependent effects on splenic lymphocytes, as revealed by flow cytometry. SAMP8 Control group exhibited a marked reduction in the total number of CD3^+^ T cells (**Figure 3A**), CD4^+^ T cells (**Figure 3B**), FoxP3^+^ Treg cells (**Figure 3D**), and CD19^+^ B cells (**Figure 3E**) compared to SAMR1 Control. An inversion of the CD4/CD8 ratio was observed between strains (**Figures 4B, C**), reflecting distinct immunological profiles. Additionally, in SAMP8 animals treated with RAPA, a significant reduction in the percentage of Treg cells was noted (**Figure 4D**).

**Figure 5:**
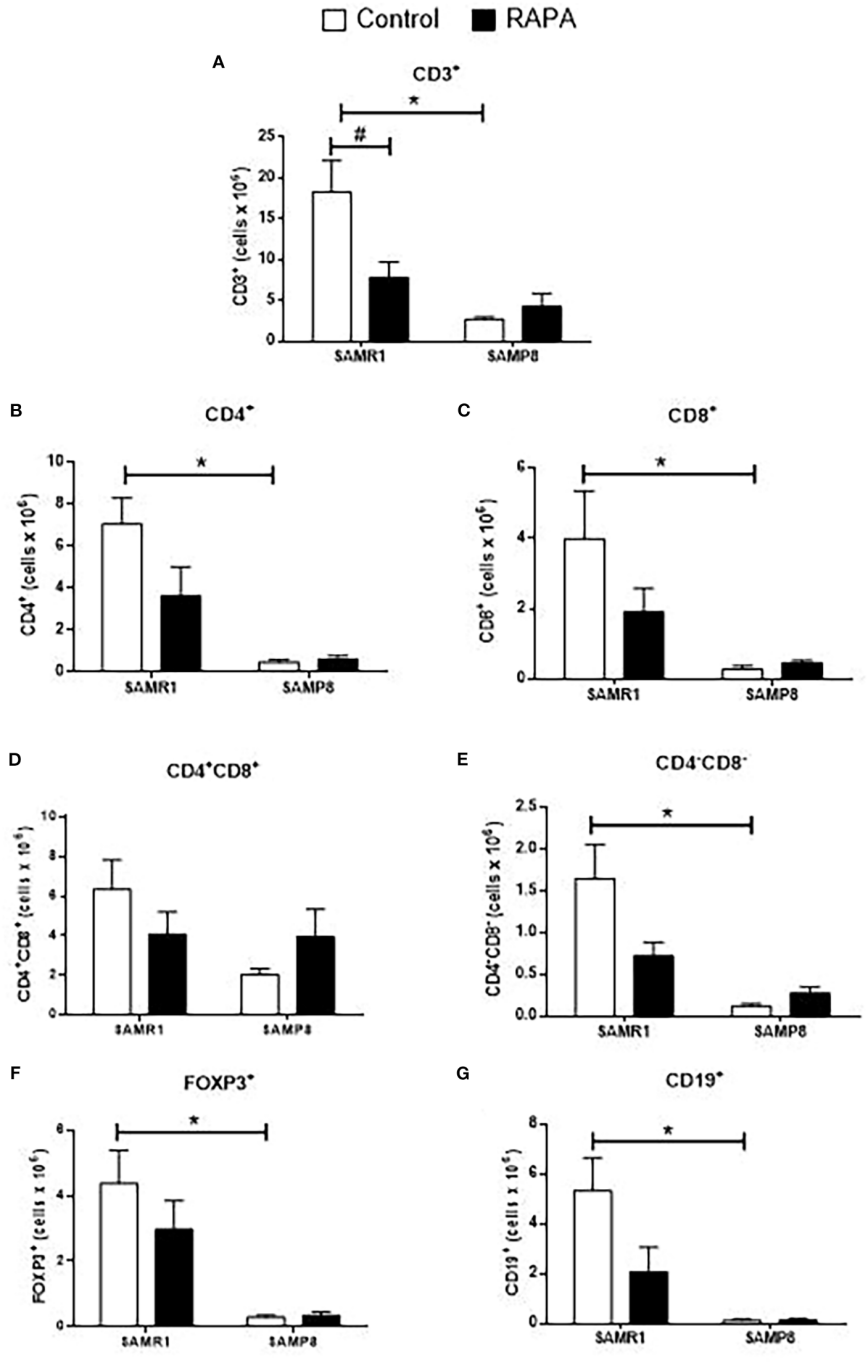
Evaluation of the total number of thymic lymphocyte populations in animals after 6 months of RAPA treatment. T lymphocytes: **(A)** Total lymphocytes (CD3^+^ cells); **(B)** T helper lymphocytes (CD4^+^ cells); **(C)** Cytotoxic T lymphocytes (CD8^+^ cells); **(D)** Double-positive T lymphocytes (CD4^+^CD8^+^ cells); **(E)** Double-negative T lymphocytes (CD4^-^CD8^-^ cells); **(F)** Regulatory T lymphocytes (FoxP3^+^ cells); B lymphocytes: **(G)** CD19^+^ cells. Results are expressed as mean ± standard error. Statistical analysis was performed using a two-way ANOVA with two fixed factors strain (SAMR1 vs. SAMP8) and treatment (Control vs. RAPA) followed by Tukey’s multiple-comparison test. *indicates a significant difference between Control strains, and # indicates a significant difference between SAMP8 groups (p ≤ 0.05). (n=4–5 animals per group). Raw data on **Supplementary Table S5**.

Evaluation of thymocytes corroborated these findings, revealing a significant reduction in the total number of most lymphocyte subpopulations in SAMP8 Control compared to SAMR1 Control animals (**Figures 5A–G**). However, no significant difference was observed in the number of CD4^+^CD8^+^ thymocytes between the Control groups (**Figure 5D**). Notably, RAPA treatment led to a reduction in total CD3^+^ T cells in SAMR1 animals compared to Controls (**Figure 5A**). A similar tendency was observed for each of the other lymphocyte populations studied, namely CD4^+^, CD8^+^, CD4^+^CD8^+^, CD4^-^CD8^-^, FoxP3^+^ Treg, and CD19^+^ cells (**Figures 5B–G**).

**Figure 6:**
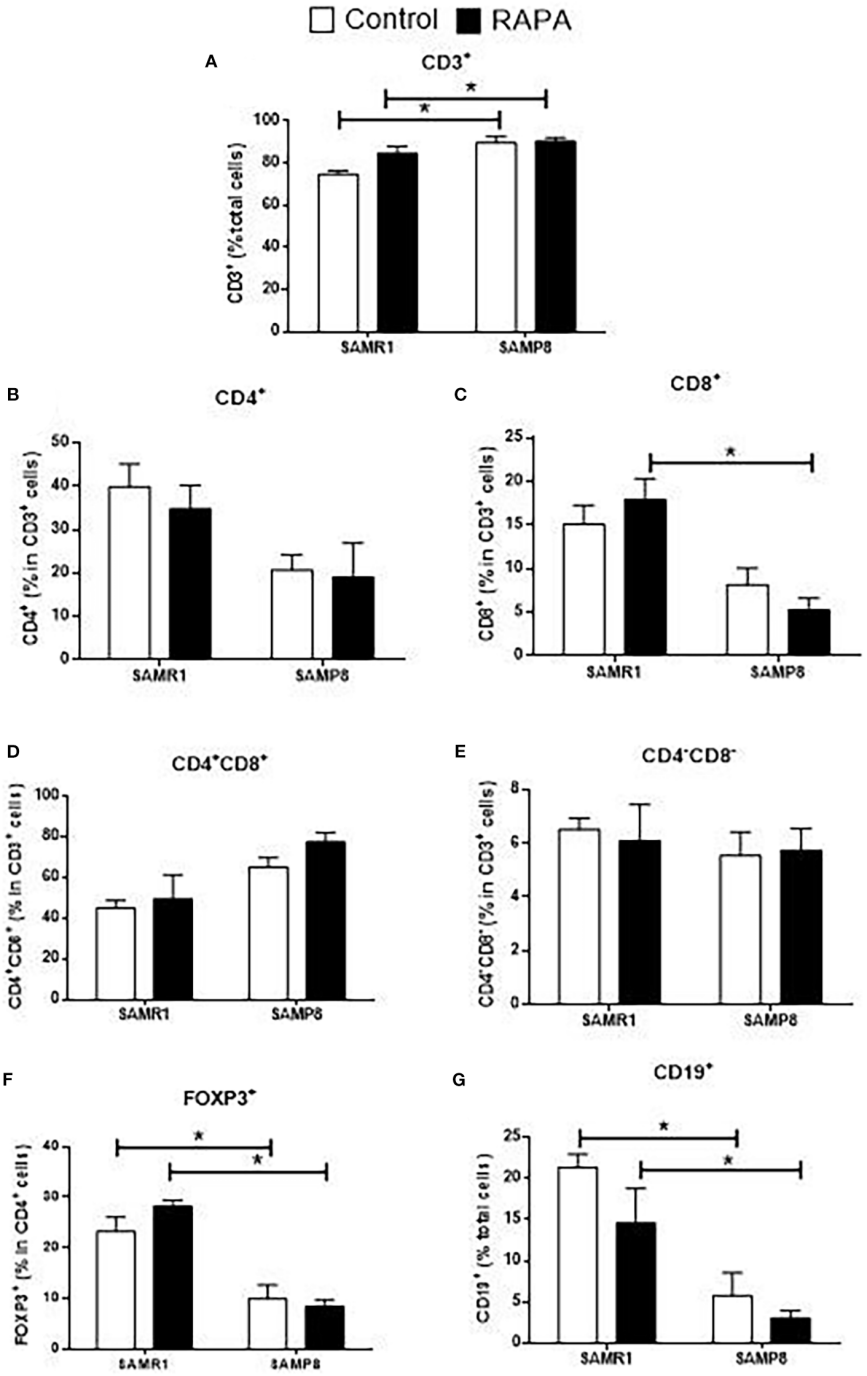
Evaluation of the percentage of thymic lymphocyte populations in animals after 6 months of RAPA treatment. **(A)** Total lymphocytes (CD3^+^ cells); **(B)** T helper lymphocytes (CD4^+^ cells); **(C)** Cytotoxic T lymphocytes (CD8^+^ cells); **(D)** Double-positive T lymphocytes (CD4^+^CD8^+^ cells); **(E)** Double-negative lymphocytes (CD4^-^CD8^-^ cells); **(F)** Regulatory T lymphocytes (FoxP3^+^ cells); B lymphocytes: **(G)** CD19^+^ cells. Results are expressed as mean ± standard error. Statistical analysis was performed using a two-way ANOVA with two fixed factors strain (SAMR1 vs. SAMP8) and treatment (Control vs. RAPA) followed by Tukey’s multiple-comparison test. *indicates a significant difference between Control strains, and # indicates a significant difference between SAMP8 groups (p ≤ 0.05). (n=4–5 animals per group). Raw data on **Supplementary Table S6**.

In the spleen, SAMP8 Control mice exhibited a reduction in the total number of CD3^+^ T cells compared with SAMR1 Controls, whereas in the thymus the percentage of CD3^+^ thymocytes was higher, as shown in **Figure 6A**, reflecting tissue-specific differences in lymphocyte distribution and maturation. For CD8^+^ cells, significance was exclusive to the RAPA-treated groups, with SAMR1 RAPA higher than SAMP8 RAPA (**Figure 6C**). SAMR1 also exhibited higher percentages of FoxP3^+^ Treg cells and CD19^+^ B cells than SAMP8 in both the Control and RAPA groups (**Figures 6F, G**). CD4^+^, double-positive (CD4^+^CD8^+^), and double-negative (CD4^-^CD8^-^) populations did not differ significantly among groups (**Figures 6B, D, E**). These results highlight the complex and strain-specific effects of RAPA on immune cell populations, with implications for its potential immunomodulatory role in aging.

### 3.4 RAPA reduces the lymphoproliferative response in SAMR1 mice

**Figure 7:**
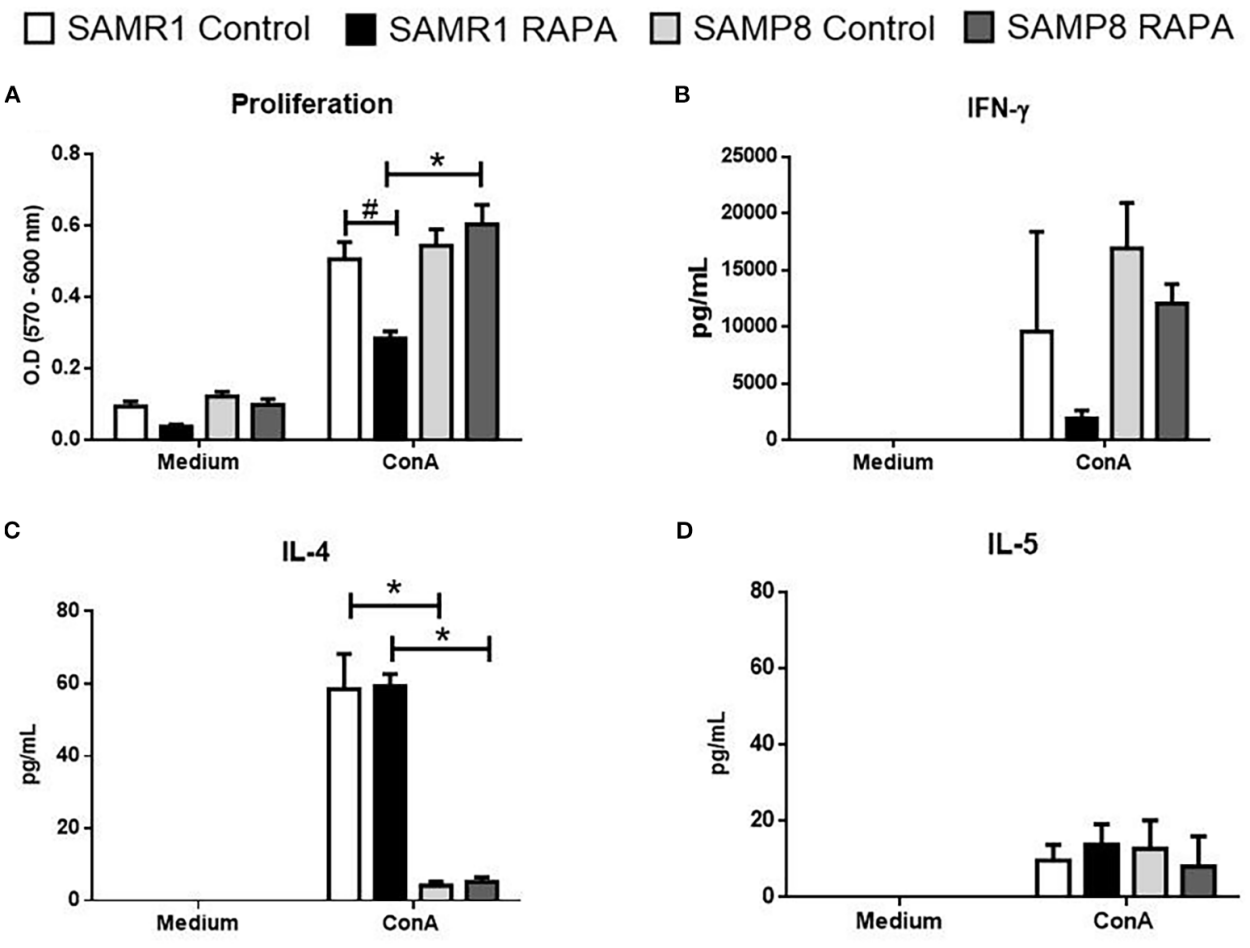
Assessment of the proliferative response and cytokine secretion of spleen lymphocytes from animals treated for 6 months with RAPA. **(A)** Lymphoproliferative response. **(B)** IFN-γ; **(C)** IL-4; and **(D)** IL-5 levels. Results are expressed as mean ± standard error. Statistical analysis was performed using a two-way ANOVA with two fixed factors strain (SAMR1 vs. SAMP8) and treatment (Control vs. RAPA) followed by Tukey’s multiple-comparison test. *indicates a significant difference between Control strains, and # indicates a significant difference between SAMP8 groups (p ≤ 0.05). (n=5 animals per group). Raw data on **Supplementary Table S7**.

The proliferative response of splenic lymphocytes was evaluated in Con A-stimulated cultures at the end of the 6 months RAPA treatment. In SAMR1 mice, RAPA treatment significantly reduced lymphocyte proliferation compared to untreated SAMR1 Control group (**Figure 7A**). Interestingly, spleen cells from SAMP8 RAPA animals exhibited higher proliferation relative to their SAMR1 RAPA counterparts, suggesting a strain-dependent modulation of immune cell activity by RAPA.

### 3.5 Spleen lymphocytes from SAMR1 mice exhibit a Th2-driven profile compared to SAMP8 lymphocytes under stimulation

Cytokine production was evaluated following *in vitro* activation of lymphocytes from animals treated with RAPA for 6 months. Among the adaptive immune mediators analyzed, IFN-γ was assessed as a representative of Th1 responses, while IL-4 and IL-5 were assessed as Th2 cytokines. As expected, none of these cytokines were produced in unstimulated cells; however, Con A stimulation induced detectable levels of all examined cytokines. The only significant difference observed was for IL-4 production, a cytokine associated with Th2 responses, which was significantly lower in spleen cells from SAMP8 Control animals compared to SAMR1 Control animals (**Figure 7B**). This finding supports a predominant Th2 profile in SAMP8 animals stimulated with Con A. Notably, RAPA treatment did not alter this profile in either strain. These results suggest that SAMP8 animals exhibit an adaptive immune response pattern characterized by reduced Th2-associated cytokine production (IL-4), potentially contributing to the immunological phenotype observed in this strain.

## 4 Discussion

The SAMP mouse strain is a valuable model for aging research due to its characteristics of accelerated senescence and a shorter lifespan (average of 9.7 months for SAMP8 *versus* 16.3 months for SAMR1) (36). The SAMP8 strain mimics many hallmarks of human aging, such as mitochondrial dysfunction and chronic inflammation, which are particularly relevant to this study’s focus on RAPA’s impact on these processes. In contrast, SAMR1 mice serve as a robust baseline for distinguishing aging-independent outcomes (37).

Aging profoundly affects the immune system, leading to immunosenescence, which in turn increases vulnerability to infections, autoimmune diseases, and cancer. Enhancing immune cell function could improve an individual’s resilience to both internal and external threats, potentially contributing to a longer lifespan. However, using RAPA as an anti-aging treatment presents a challenge due to its immunosuppressive properties, which inhibit cell proliferation by blocking mTOR. In their study, Arriola Apelo et al. (38) found that intermittent administration of RAPA at 2 mg/kg every five days minimized adverse effects. Based on these results, we employed a similar regimen to investigate whether low-dose, intermittent treatment could ameliorate RAPA-induced immune and metabolic alterations.

Phenotypes of immunosenescence include thymic atrophy, reduced numbers of naïve T cells, increased numbers of memory T cells, and a reversal of the CD4/CD8 ratio, all of which directly influence immune responses in aged individuals (13). In the study by Neff et al. (2013), C57BL/6 mice received encapsulated RAPA at 14 ppm (approximately 2.24 mg/kg/day), and analysis of peripheral blood lymphocyte subpopulations in young and aged animals showed that the frequency of CD4^+^ T lymphocytes declined with age in both Control and RAPA-treated groups. Importantly, RAPA treatment did not reverse this age-associated decline in CD4^+^ T cells (39). This pattern closely parallels our findings in the accelerated aging SAMP8 mice, where a reduction in CD4^+^ lymphocytes was observed in both the thymus and spleen compared to the SAMR1 strain. Similarly, RAPA treatment at a low dose was not able to prevent or restore this loss. These results underscore the importance of examining RAPA’s effects on both primary and secondary lymphoid organs, as well as on *in vitro* lymphocyte proliferation, to better understand its influence on immune aging.

Comparative studies evaluating the effects of RAPA across primary and secondary lymphoid organs highlight distinct tissue-specific responses. For example, Luo et al. (1994) demonstrated that BALB/c and DBA/2 mice treated intraperitoneally with 2 mg/kg RAPA for 14 days developed significant thymic atrophy and decreased thymocyte proliferation, yet spleen mass and cellularity remained unaffected (40). Similarly, Tian et al. (2004) found that a regimen of 1.5 mg/kg RAPA i.p. for 14 days in Wistar rats accelerated apoptosis in thymic CD4^+^CD8^+^ thymocytes but did not decrease splenic cell numbers, although it increased the proportion of CD4^+^CD25^+^ T cells, suggesting functional but not quantitative alterations (41). Lu et al. (2015) further demonstrated that only higher chronic doses of RAPA (1–3 mg/kg/day) reduced cellularity in the thymic and spleen of young rats, whereas lower doses had milder effects (42). In our previous work, we showed that 2 months of low-dose RAPA treatment induced more rapid thymic involution in SAMP8 mice, which exhibited significantly lower thymic cellularity than SAMR1. Importantly, RAPA treatment did not promote thymic atrophy in these animals. In contrast, in the spleen, SAMP8 mice displayed increased CD4^+^ and decreased CD8^+^ splenic T cells, while SAMR1 mice showed the opposite effect (31). However, these alterations were not sustained during chronic exposure, as observed in the present study.

Qu et al. (2007) reported that 1.5 mg/kg/day of RAPA i.p. for 14 days in C57BL/6 mice increased the proportion of splenic CD4^+^CD25^+^FoxP3^+^ Treg cells, despite a reduction in total lymphocyte numbers, indicating differential sensitivity of Treg cells to RAPA (16). In our own study, we observed a reduction in Treg cell percentages in SAMP8 RAPA animals, while SAMR1 RAPA animals showed decreased thymic CD3^+^ cellularity and diminished *in vitro* lymphoproliferative capacity. These observations align with the partial resistance of Treg cells to RAPA reported by Wang et al. (2011), who used 1.25 mg/kg daily or 3 mg/kg on alternate days in C57BL/6 mice (43), as well as with the broader variability seen in long-term regimens, such as Hurez et al. (2015) using 2.24 mg/kg/day encapsulated RAPA for 12 months (44).

It is important to note that RAPA administration may impact gut microbiota, as shown in recent studies. Hurez et al. (2015) demonstrated that RAPA can modify microbial composition, which may in turn influence immune function, given the bidirectional nature of the gut–immune axis (44). Although our study focused on direct effects of RAPA on glucose metabolism and immune cells, we recognize that the absence of microbiota monitoring is a limitation. Future investigations incorporating gut microbiota analysis will be crucial to fully elucidate the mechanisms underlying the systemic effects of RAPA.

In our study, we observed a reduction in the proportion of FoxP3^+^ Tregs in SAMP8 mice treated with RAPA. Although previous reports (41) indicate that RAPA may accelerate apoptosis in CD4^+^CD8^+^ thymocytes, our methodology, by flow cytometry with LIVE/DEAD™ viability staining, did not allow us to determine whether the observed decrease in FoxP3^+^ cells reflects increased apoptosis, suppressed proliferation, or both.

Beyond quantitative changes, the decrease of CD3^+^ thymocytes and altered CD4/CD8 ratios observed here have broader functional implications in the context of immunosenescence. Thymic atrophy and reduced naïve T cell output are hallmarks of aging, contributing to diminished repertoire diversity and impaired responses to novel antigens (45). Our findings suggest that intermittent RAPA, despite its mitochondrial benefits, may accentuate some of these age-associated immune limitations. This raises important translational considerations, as any clinical use of RAPA in elderly populations would need to balance its anti-aging benefits with potential risks of reduced immune competence.

A study by Aoki et al. (1995) (46) analyzed SAMP8 mice at 9 months of age, which is the same age as the animals in our study at the time of analysis. In their study, SAMP8 mice exhibited increased IFN-γ and decreased IL-2, IL-4, and IL-5 compared to the background AKR/J strain. This was attributed to immunosenescence in SAMP8 mice, resulting in an inflammatory profile and reduced IL-4 production associated with poor antibody responses. While our results differed from Aoki et al. (1995) for most cytokines, both studies consistently showed a significant decrease in IL-4 in SAMP8, reinforcing its association with immune aging and poor antibody responses in this strain. Collectively, these findings underscore that the effects of RAPA on immune aging are shaped by protocol, dosage, animal strain, and underlying immune phenotype.

In addition to differences in inflammatory profiles, aging-related metabolic changes, such as altered glucose uptake, offer further insights into the physiological distinctions between SAMP8 and SAMR1 strains. A crucial aspect of *in vivo* glucose metabolism is cellular responsiveness to insulin, which is frequently impaired with advancing age. Notably, prolonged RAPA treatment has been associated with the development of insulin resistance and hyperglycemia (39). To address these concerns, we adopted the intermittent dosing regimen proposed by Arriola Apelo et al. (2016) (38), in which RAPA is administered every five days, a strategy shown to effectively mitigate both glucose-related and immunosuppressive side effects. Indeed, in our results, no difference in glucose metabolism was observed following RAPA treatment, except for reduced glucose uptake in the bladder of SAMP8 RAPA animals. The apparent reduction in bladder ^18^F-FDG signal in SAMP8 animals treated with RAPA likely reflects urinary excretion rather than metabolic uptake, as the bladder does not actively metabolize glucose. This secondary observation may be linked to renal physiology or urine output and should not be overinterpreted as an effect on systemic glucose metabolism. Furthermore, our results demonstrate that SAMP8 Control animals exhibit a higher basal glucose demand compared to SAMR1 Control, likely attributable to oxidative stress, which can activate compensatory glucose uptake pathways even under low-glucose conditions. This is consistent with the *ex vivo* findings of Barquissau et al. (2017) (47), who reported increased glucose uptake in muscle from SAMP8 and SAMR1 mice under overnight fasting. Our current study extends these observations to the *in vivo* context.

Consistent with prior evidence of a chronic low-grade inflammatory state in SAMP8 mice, which is associated with increased oxidative stress and metabolic reprogramming (48, 49), the elevated basal glucose uptake we observed may reflect compensatory glucose transport and mitochondrial substrate use rather than enhanced adaptive immune activity. This supports the concept of inflammaging, where metabolic and immune alterations are partially uncoupled.

Finally, the bioenergetic profile analysis of liver mitochondria revealed no statistical differences in mitochondrial respiration between strains, contrary to expectations that accelerated aging drives oxidative stress and energy production inefficiency (50). This finding can be explained by biological factors: all animals were approximately 9 months old at the end of the experiment, an age at which SAMP8 mice may not yet display overt mitochondrial dysfunction in the liver (51). Moreover, SAMP8 is primarily a model of sarcopenia, and it is well recognized that different organs age at different rates. The liver, in particular, has a high regenerative capacity and cellular turnover, which may delay or attenuate mitochondrial impairment compared with more aging-sensitive tissues such as skeletal muscle (52, 53). However, RAPA improved ATP synthesis in state 3 of mitochondrial respiratory in treated animals from both strains. Similar beneficial effects of RAPA have been observed in skeletal muscle (54), cardiac muscle (55), and even at low doses (0.8 mg/kg) in the muscle and brain of mice (56). This improvement can be attributed to RAPA’s role in promoting mitophagy, a crucial process for mitochondrial quality control, a mechanism we did not directly investigate in this study. Furthermore, our results showed that RAPA treatment enhanced respiratory control in SAMR1 animals, indicating improved mitochondrial efficiency in oxygen utilization. This finding aligns with previous research, such as that by Johnson et al. (2013) in a mouse model of Leigh syndrome treated with RAPA (57).

Although our data demonstrate improved mitochondrial bioenergetics and distinct strain-specific immune outcomes, the mechanistic basis of these effects remains to be fully elucidated. Our interpretation that RAPA promotes mitophagy is hypothesis-driven and relies on indirect evidence from the literature, as we did not measure canonical markers of mitophagy such as PINK1/Parkin activation, LC3-II turnover, or mitochondrial DNA integrity. Likewise, the strain-specific immune responses observed, such as reduced FoxP3^+^ Tregs in SAMP8 and decreased CD3^+^ thymocytes in SAMR1, may reflect differences in basal mTORC activity, IL-2 signaling, or epigenetic regulation that were not assessed here. Future investigations integrating direct mitophagy assays, detailed cytokine profiling, and epigenetic analyses will be critical to define the molecular drivers of these RAPA effects and to refine therapeutic strategies based on mTOR modulation.

These results demonstrate that RAPA treatment exerts distinct effects between the strains, impacting not only the immune system but also mitochondrial bioenergetics. These differences may be attributable to a relatively preserved mitochondrial function and metabolic integrity in SAMR1 animals, which experience normal aging and likely exhibit less age-associated cellular damage compared to SAMP8. SAMR1 mice showed a more pronounced immunosuppressive response and greater mitochondrial adaptability to RAPA, which may be due to a higher baseline capacity for bioenergetic and metabolic regulation. This enhanced responsiveness could reflect more efficient mitochondrial dynamics, energy production, and cellular signaling pathways that are possibly maintained in SAMR1 but compromised in SAMP8 as a result of accelerated aging. These findings underscore the need for further research to clarify the mechanisms underlying these strain-specific responses and to explore the broader implications of RAPA treatment in modulating mitochondrial bioenergetics, immune function, and aging-related processes.

While our study provides novel insights into the long-term effects of intermittent low-dose RAPA, several limitations should be acknowledged. On the immune side, analyses were restricted to lymphocyte subsets, proliferative responses, and a limited cytokine panel, without *in vivo* tests of immune competence (e.g., infection or vaccination models) or functional characterization of specific subsets such as the suppressive capacity of FoxP3^+^ Tregs, antigen-specific proliferation, cytotoxic activity, or effector cytokine production at the single-cell level. On the metabolic side, although ^18^F-FDG PET enabled monitoring of tissue glucose handling, we did not perform insulin or glucose tolerance tests, indirect calorimetry, or detailed metabolic flux analyses needed to fully define the adaptive nature of RAPA’s effects. Furthermore, although SAMP8 mice are widely used as a model of accelerated aging, we did not include direct measures of lifespan, healthspan, or age-related pathologies, and immunosenescence was inferred primarily from changes in lymphocyte subsets and cytokine production without validation against canonical senescence markers or broader SASP components. In addition, baseline molecular differences in mTOR signaling, mitochondrial function, or immune homeostasis were not directly assessed, and the absence of significant differences in liver mitochondrial respiration between strains likely reflects the true metabolic status of 9-month-old animals, when SAMP8 typically exhibit only moderate hepatic mitochondrial impairment and the liver’s high regenerative capacity may delay dysfunction, rather than methodological artifacts. Taken together, these limitations indicate that our findings provide indirect but meaningful evidence of age-associated immune and metabolic modulation. Future studies combining functional immune assays, comprehensive senescence and SASP marker profiling, and longitudinal aging outcomes, as well as testing combinatorial strategies with immunomodulatory adjuvants or metabolic regulators, will be critical to validate and expand the translational implications of chronic intermittent low-dose RAPA.

## 5 Conclusions

The findings presented here confirm the potential of RAPA as a modulator of aging-related processes when administered chronically at intermittent low doses, as evidenced by improvements in mitochondrial function and energy metabolism observed across different strains. However, the immunosuppressive effects of RAPA, including reductions in Treg percentages and total CD3^+^ T lymphocytes in SAMP8 and SAMR1, respectively, highlight the challenge of balancing efficacy and safety in therapeutic applications. These results lay the groundwork for refining RAPA-based interventions by focusing on the optimization of dosing regimens to effectively target specific hallmarks of aging while minimizing systemic effects. Further research is essential to tailor these interventions, improve the therapeutic index, and ensure more precise modulation of the aging process.

## Data Availability

The raw data supporting the conclusions of this article will be made available by the authors, without undue reservation.

## Supplementary material

Supplementary Figure 1 Flow cytometry gating strategy. First, lymphocytes were selected by size and granularity (FSC-A versus SSC-A), followed by exclusion of doublets using FSC-H versus FSC-A and SSC-H versus SSC-A plots. Next, live cells were identified by negative staining with the LIVE/DEAD™ Aqua viability dye to ensure inclusion of only viable singlets. Within this live lymphocyte gate, CD3^+^ T cells and CD19^+^ B cells were identified, and the CD3^+^ population was further subdivided into CD4^+^ T helper, CD8^+^ cytotoxic, and FoxP3^+^ regulatory T cells using fluorescence-minus-one (FMO) Controls to establish boundaries and confirm specificity. For thymus samples, additional gating allowed discrimination of CD4^+^CD8^+^ double-positive and CD4^-^CD8^-^ double-negative subsets within the CD3^+^ compartment. This integrated and sequential approach ensured reproducibility, minimized false-positive events, and provided robust quantification of all lymphocyte subpopulations analyzed.

Supplementary Table 1 Raw data of **Figure 1**. Data represent individual animal measurements used to generate **Figure 1**.

Supplementary Table 2 Raw data of **Figure 2**. Data represent individual animal measurements used to generate **Figure 2**.

Supplementary Table 3 Raw data of **Figure 3**. Data represent individual animal measurements used to generate **Figure 3**.

Supplementary Table 4 Raw data of **Figure 4**. Data represent individual animal measurements used to generate **Figure 4**.

Supplementary Table 5 Raw data of **Figure 5**. Data represent individual animal measurements used to generate **Figure 5**.

Supplementary Table 6 Raw data of **Figure 6**. Data represent individual animal measurements used to generate **Figure 6**.

Supplementary Table 7 Raw data of **Figure 7**. Data represent individual animal measurements used to generate **Figure 7**.
