# Supplementary figures and images for: Effects of Long-term low-dose intermittent rapamycin administration on glucose metabolism and immune system of SAMP8 and SAMR1 mice
Source: Front Immunol. 2025 Oct 21;16:1682406. doi: 10.3389/fimmu.2025.1682406 (PMC12598397; doi:10.3389/fimmu.2025.1682406)

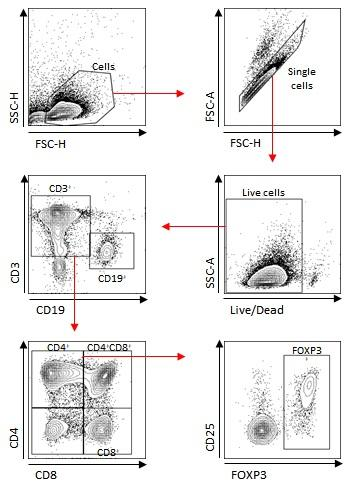

Supplement: Supplementary Figure 1 — Flow cytometry gating strategy. First, lymphocytes were selected by size and granularity (FSC-A versus SSC-A), followed by exclusion of doublets using FSC-H versus FSC-A and SSC-H versus SSC-A plots. Next, live cells were identified by negative staining with the LIVE/DEAD™ Aqua viability dye to ensure inclusion of only viable singlets. Within this live lymphocyte gate, CD3+ T cells and CD19+ B cells were identified, and the CD3+ population was further subdivided into CD4+ T helper, CD8+ cytotoxic, and FoxP3+ regulatory T cells using fluorescence-minus-one (FMO) Controls to establish boundaries and confirm specificity. For thymus samples, additional gating allowed discrimination of CD4+CD8+ double-positive and CD4-CD8- double-negative subsets within the CD3+ compartment. This integrated and sequential approach ensured reproducibility, minimized false-positive events, and provided robust quantification of all lymphocyte subpopulations analyzed. [file Image1.tiff]
