# Supplementary material for: Effects of Long-term low-dose intermittent rapamycin administration on glucose metabolism and immune system of SAMP8 and SAMR1 mice
Source: Front Immunol. 2025 Oct 21;16:1682406. doi: 10.3389/fimmu.2025.1682406 (PMC12598397; doi:10.3389/fimmu.2025.1682406)
Supplement: Supplementary Table 1 — Raw data of Figure 1 . Data represent individual animal measurements used to generate Figure 1 . [file DataSheet1.docx]

**Supplementary Figure 1.** Flow cytometry gating strategy. First, lymphocytes were selected by size and granularity (FSC-A versus SSC-A), followed by exclusion of doublets using FSC-H versus FSC-A and SSC-H versus SSC-A plots. Next, live cells were identified by negative staining with the LIVE/DEAD™ Aqua viability dye to ensure inclusion of only viable singlets. Within this live lymphocyte gate, CD3⁺ T cells and CD19⁺ B cells were identified, and the CD3⁺ population was further subdivided into CD4⁺ T helper, CD8⁺ cytotoxic, and FoxP3⁺ regulatory T cells using fluorescence-minus-one (FMO) Controls to establish boundaries and confirm specificity. For thymus samples, additional gating allowed discrimination of CD4⁺CD8⁺ double-positive and CD4⁻CD8⁻ double-negative subsets within the CD3⁺ compartment. This integrated and sequential approach ensured reproducibility, minimized false-positive events, and provided robust quantification of all lymphocyte subpopulations analyzed.

**Supplementary Table S1.** Raw data of Figure 1. Data represent individual animal measurements used to generate Figure 1.

**Supplementary Table S2.** Raw data of Figure 2. Data represent individual animal measurements used to generate Figure 2.

**Supplementary Table S3.** Raw data of Figure 3. Data represent individual animal measurements used to generate Figure 3.

**Supplementary Table S4.** Raw data of Figure 4. Data represent individual animal measurements used to generate Figure 4.

**Supplementary Table S5.** Raw data of Figure 5. Data represent individual animal measurements used to generate Figure 5.

**Supplementary Table S6.** Raw data of Figure 6. Data represent individual animal measurements used to generate Figure 6.

**Supplementary Table S7.** Raw data of Figure 7. Data represent individual animal measurements used to generate Figure 7.
